# Rectal Cancer—Report of Symposium

**Published:** 1985-01

**Authors:** N. J. McC. Mortensen

**Affiliations:** Consultant Surgeon and Senior Lecturer, Bristol Royal Infirmary


### Rectal Cancer – Operative Strategy and Prevention of Recurrence

### Report on a Symposium at the Edward Jenner Centre, November 1984.

**N. J. McC. Mortensen MD FRCS**
Consultant Surgeon and Senior Lecturer, Bristol Royal Infirmary

Rectal cancer accounts for 5% of all deaths from cancer, and 10,000 new cases present each year. The overall five-year survival is 31% and one of the most distressing forms of recurrence after surgery is local recurrence. What is surprising about this local recurrence is that it varies from surgeon to surgeon by from 5 to 35%.[1] There is also the suspicion that with the introduction of stapling devices and increasing numbers of restorative procedures this local recurrence may be on the increase. So the patient with a cancer of the middle third of the rectum (up to 12 cm from the anal verge) is in the eye of a storm of controversy – should he have an abdomino-perineal resection and a life long colostomy or risk local recurrence with a restorative procedure? Whilst a 5 cm distal clearance from the tumour was widely accepted, a compromise of as little as 1 to 2 cm clearance does not appear to influence local recurrence.[2]

The surgical options will depend upon the distance of the rectal tumour from the anal verge, the physical features of the tumour including the numbers of quadrants involved and mobility, the histological grade of the preoperative biopsy and the presence or absence of metastases. Preoperative staging by digital examination and CT scanning will accurately predict involvement of adjacent organs by major local spread but not minor degrees of local invasion.[3] Rectal ultrasound may be useful but has not yet been assessed in clinical use.

Mr. Umpleby (Senior Surgical Registrar, Bath) presented a review of the evidence for local recurrence occurring by tumour cell implantation at the time of the anastomosis. There is experimental work which demonstrates that viable colon cancer cells can be recovered from the colonic lumen considerable distances away from the tumour. These cells are not yet demonstrably clonogenic, but there is sufficient evidence to warrant routine irrigation of the rectal stump with a cytocidal agent before dividing the rectum – a procedure which only 19% of surgeons in the South West employ.[4]

Mr. C. Marks (Consultant Surgeon, Guildford) reported the results of a new clearance technique for lymph nodes in resection specimens for rectal cancer. Lymph node involvement by Dukes classification has a major impact on prognosis and its detection will depend upon the number of lymph nodes examined by the histopathologist. Standard histopathology preparation and reporting is also crucial to the careful comparison of results from different centres. In this technique the specimen was pinned out, the mesorectum removed and the fat removed by immersion in xylol. Significantly more lymph nodes were identified for examination using this method than either at St. Marks or a number of other non-specialist hospitals.

Dr. V. Barley (Consultant Radiotherapist, Bristol Royal Infirmary) reviewed the place of radiotherapy in the treatment of rectal cancer which is still secondary to primary surgical treatment. Trials and reports on adjuvant preoperative radiotherapy have demonstrated a reduction in the numbers of C cases but no increase in survival. The recent MRC trial was disappointing in not showing any clear benefit for radiotherapy. Fixed tumours may be made operable by radiotherapy and a further MRC trial is in progress. Postoperative adjuvant radiotherapy for B and C cases may have a place, but this is not yet proven, but it is hoped that local recurrence will be reduced.[5]

Mr. R. J. Heald (Consultant Surgeon, Basingstoke) described the background to his personal series of over 150 cases of rectal cancer. He has an enviably low local recurrence rate, but ascribes this to meticulous dissection and excision of all the mesorectum.[6] Local recurrence after restorative resection was due to failure to remove micro invasion in lymphatics within the mesorectum distal to the tumour. Middle and lower third cancers should always be excised together with the entire mesorectum, and the distal bowel clearance is less important. More radical surgery including iliac lymphadenectomy was unnecessary and damaged important pelvic nerves.[7]

Rectal cancer is a common surgical problem managed by a range of general surgeons. The symposium was an important focus for careful consideration of the factors likely to either lead to or prevent local recurrence.

### REFERENCES

1. PHILLIPS, R. K. S., HITTINGER, R., BLESOVSKY, L., FRY, J. S., FIELDING, C. P. (1984) Local recurrence after curative surgery for large bowel cancer (1) the overall picture. *Br. J. Surg.* **71**, 12–16.
2. WILLIAMS, N. S. (1984) The rationale for preservation of the anal sphincter in patients with low rectal cancer. *Br. J. Surg.* **71**, 575–581.
3. NICHOLLS, R. J., YORK MASON, A., MORSON, B. C., DIXON, A. D., KELSEY FRY, I. (1982) The clinical staging of rectal cancer. *Br. J. Surg.* **69**, 404–409.
4. UMPLEBY, H. C., FERMOR, B., SYMES, M. O., WILLIAMSON, R. C. N. (1984) Viability of exfoliated colorectal carcinoma cells. *Br. J. Surg.* **71**, 659–663.
5. SISCHY, B. (1982) The place of radiotherapy in cancer of the rectum. *Cancer* **50**, 2631.
6. HEALD, R. J., HUSBAND, E. M., RYALL, R. D. M. (1982) The mesorectum in rectal cancer surgery – the clue to rectal pelvic recurrence? *Br. J. Surg.* **69**, 613–616.
7. EDITORIAL (1983) Conserving the sphincters in rectal cancer. *Lancet* **ii**, 717–718.

